# Lockdowns due to COVID-19 threaten PhD students’ and early-career researchers’ careers

**DOI:** 10.1038/s41559-020-1231-5

**Published:** 2020-06-03

**Authors:** José Ricardo Paula

**Affiliations:** MARE – Marine and Environmental Sciences Centre, Laboratório Marítimo da Guia, Faculdade de Ciências, Universidade de Lisboa, Cascais, Portugal

**Keywords:** Ecology, Evolution, Scientific community, Science, technology and society

**To the Editor** — The lockdowns to contain the current COVID-19 pandemic could unduly impact PhD students’ and early-career researchers’ careers. This is due to the vulnerability of their income, and the time-constrained nature of student and early-career researcher (ECR) research programmes. Many PhD students rely on fellowships that can span from one to four years. These students might be affected directly in their effective scholarship time and have to pay extra tuition fees. Early-career researchers (that is, post-docs) also rely on short-duration contracts (usually one to two years) and are expected to produce high outputs. A long break in their contracts could jeopardize their ability to complete the research program they proposed, compromising future job applications. For students and ECRs in ecology and evolution fields, the inability to conduct field or laboratory work essential to their studies may exacerbate these problems. We need to implement solutions for the ecology and evolution community during this pandemic. These could include the extension of PhD fellowships for the duration of lockdown. For example, in Portugal, the national science foundation (FCT) has extended all fellowships for two months, and further extensions are in consideration if the situation continues^1,2^. However, this measure should be followed by a temporary suspension of tuition fees payments as such extensions imply a higher PhD enrolment time and might result in extra fees. In order for students to progress to the next stage of their careers, universities will need to provision remote thesis defences, but with enough security to prevent internet hijacking (also known as ‘zoombombing’)^3^. For early-career researchers, funding agencies and institutes must consider the extension of their contracts to account for the effect of this break.

It is also essential to understand that being locked down at home does not equate to a boost in productivity. We are living through stressful times, and even if our work can be performed remotely, other concerns, such as caring for family and coping with mental and physical health, must take precedence and will affect productivity.
